# "I thought I was going to die": Experiences of COVID-19 patients managed at home in Uganda

**DOI:** 10.1371/journal.pone.0295113

**Published:** 2023-12-12

**Authors:** Susan Nakireka, David Mukunya, Crescent Tumuhaise, Ronald Olum, Edith Namulema, Agnes Napyo, Quraish Serwanja, Prossie Merab Ingabire, Asad Muyinda, Felix Bongomin, Milton Musaba, Vivian Mutaki, Ritah Nantale, Phillip Akunguru, Rozen Ainembabazi, Derrick Nomujuni, William Olwit, Aisha Nakawunde, Specioza Nyiramugisha, Pamela Mwa Aol, Joseph Rujumba, Ian Munabi, Sarah Kiguli

**Affiliations:** 1 Department of Internal Medicine, Mengo Hospital, Kampala, Uganda; 2 Department of Internal Medicine, Uganda Christian University, School of Medicine, Mukono, Uganda; 3 Department of Community and Public Health, Faculty of Health Sciences, Busitema University, Mbale, Uganda; 4 Department of Research, Nikao Medical Center, Kampala, Uganda; 5 Department of Medicine, Our Lady Health of the Sick, Nkozi Hospital, Mpigi, Uganda; 6 Department of Medicine, St. Francis Hospital Nsambya, Kampala, Uganda; 7 School of Public Health, Makerere University, Kampala, Uganda; 8 Department of Public Health, Faculty of Health Sciences, Uganda Martyrs University, Kampala, Uganda; 9 Department of Programmes, Relief International, Khartoum, Sudan; 10 Department of Medicine, Jinja Regional Referral Hospital, Jinja, Uganda; 11 Department of Medical Microbiology, Faculty of Medicine, Gulu University, Gulu, Uganda; 12 Department of Obstetrics and Gynecology, Faculty of Health Sciences, Busitema University, Mbale, Uganda; 13 Department of Medicine, Moroto Hospital, Moroto, Uganda; 14 Department of Medicine, Jaro Hospital, Wakiso, Uganda; 15 Department of Medicine, Mulago Hospital, Kampala, Uganda; 16 Department of Radiology, Uganda Cancer Institute, Kampala, Uganda; 17 Department of Pediatrics, Lacor Hospital, Gulu, Uganda; 18 Department of Anatomy, School of Medicine, Makerere University, Kampala, Uganda; 19 Department of Pediatrics and Child Health, School of Medicine, Makerere University, Kampala, Uganda; Sefako Makgatho Health Sciences University, SOUTH AFRICA

## Abstract

**Background:**

In Uganda, approximately 170,000 confirmed COVID-19 cases and 3,630 deaths have been reported as of January 2023. At the start of the second COVID-19 wave, the Ugandan health system was overwhelmed with a sudden increase in the number of COVID-19 patients who needed care, and the Ministry of Health resorted to home-based isolation and care for patients with mild to moderate disease. Before its rollout, the COVID-19 home-based care strategy had neither been piloted nor tested in Uganda.

**Objective:**

To explore the experiences of COVID-19 patients managed at home in Uganda.

**Methods:**

This was a qualitative study that was conducted to explore the lived experiences of COVID-19 patients managed at home. The study was carried out among patients who presented to three hospitals that were designated for treating COVID-19 patients in Uganda. COVID-19 patients diagnosed at these hospitals and managed at home were followed up and contacted for in-depth telephone interviews. The data were analysed using thematic content analysis with the aid of NVIVO 12.0.0 (QRS International, Cambridge, MA).

**Results:**

Participants experienced feelings of fear and anxiety: fear of death, fear of losing jobs, fear of infecting loved ones and fear of adverse events such as loss of libido. Participants also reported feelings of loneliness, hopelessness and depression on top of the debilitating and sometimes worsening symptoms. In addition to conventional medicines, participants took various kinds of home remedies and herbal concoctions to alleviate their symptoms. Furthermore, COVID-19 care resulted in a high economic burden, which persisted after the COVID-19 illness. Stigma was a major theme reported by participants. Participants recommended that COVID-19 care should include counselling before testing and during and after the illness to combat the fear and stigma associated with the diagnosis. Another recommendation was that health workers should carry out home visits to patients undergoing home-based care and that COVID-19 treatment should be free of charge.

**Conclusion:**

COVID-19 home-based care was associated with fear, anxiety, loneliness, depression, economic loss and stigma. Policymakers should consider various home-based follow-up strategies and strengthen counselling of COVID-19 patients at all stages of care.

## Introduction

COVID-19 has so far resulted in over 690 million confirmed cases and more than 6.9 million deaths globally as of November 2023 [1]. In Uganda, approximately 170,000 confirmed COVID-19 cases and 3,630 deaths have been reported as of November 2023 [1]. The first case of COVID-19 in Uganda was detected on 21^st^ March 2020 [2]. In November 2020, Uganda experienced an increase in the number of critically ill patients (first COVID-19 wave (November to December 2020)). This coincided with the emergence of the delta variant in South Africa. During this period, the Ministry of Health updated the public through media on the prevailing COVID-19 situation, but there was a lot of skepticism. Between April and June 2021, there was a resurgence of COVID-19 cases (second COVID-19 wave). Given the number of deaths in the community, the second wave was treated with greater concern and less skepticism compared to the first wave. Community COVID-19 task forces were also revived.

Unlike the first wave, health facilities were overwhelmed by a sudden increase in the number of COVID-19 patients who needed care. To ease the burden on the health facilities, Uganda’s Ministry of Health resorted to home-based isolation and care for patients with mild to moderate disease [3]. This was in contrast to the protocols in the first wave that mandated health facility treatment and isolation for all COVID-19 patients [4]. During home-based isolation, patients were required to stay in separate rooms from other family members and follow infection and prevention control guidelines [3]. They also had to agree to be monitored by health workers for two weeks and to be admitted to the hospital in case their symptoms worsened. In addition, patients were provided with a daily symptom monitoring tool, a USSD code by the Ministry of Health (*260#), for reporting and were to be followed up by health workers either in person or over the phone [3]. However, there is reason to believe that many of the assumptions about home-based care were not supported by evidence. No formal assessment has been conducted to determine the success of a home-based isolation and care strategy in Uganda.

Many homesteads in low-income settings cannot afford the isolation of COVID-19 patients due to space limitations. In addition, the referral and follow-up systems are still poorly developed to deal with COVID-19 emergencies [5]. Furthermore, it is not yet known whether adequate follow-up of patients managed at home with COVID-19 was done. We have not yet come across any published qualitative literature on the experiences of COVID-19 patients who have undergone home-based care in a similar setting. Studies conducted elsewhere have found that patients managed at home experience anger, guilt, shame, anxiety, stigma and ambiguity during their disease [6–8]. Stigma has been documented in relation to COVID-19, with fear of infection, misinformation, economic consequences of the disease, lack of awareness, and socially constructed stereotypes as common drivers [9]. However, the experience of stigma and how patients with COVID-19 cope (respond) have not been studied in the context of home-based care settings. Exploring and understanding the experiences of patients with COVID-19 managed at home will generate contextual information on how to deal with future outbreaks [10]. The use of patients’ experiences is in line with the world health organisation’s recommendation to incorporate patient voices in policy-making [11]. One of the key weaknesses of existing COVID-19 policies is the absence of patients’ voices [12].

Against this background, we aimed to explore the experiences of COVID-19 patients managed at home in Uganda. We borrowed Erving Goffman’s theory of stigma to interpret our findings. Erving describes stigma as the ’situation of an individual who is disqualified from full social acceptance’. Goffman posits that stigma is created when an individual acquires an identity that a given society views as unacceptable (spoiled identity) [13]. As such, stigma occurs as a discrepancy between how a person is characterised by society and the attributes possessed by a person [13]. Scambler categorised stigma into felt stigma and enacted stigma [14]. Felt stigma (internal stigma or self-stigmatisation) refers to the shame and expectation of discrimination that prevents people from talking about their experiences and stops them from seeking help. Enacted stigma (external stigma, discrimination) refers to the experience of unfair treatment by others [14]. The theory of stigma has been widely used to understand the experience of stigma with other pandemics, including HIV [15–18] and, more recently, Ebola [19, 20]. Our choice of the stigma theory was further premised on COVID-19 being a relatively new disease, with many unknowns and uncertainties which could give rise to a fear that can, in turn, fuel stigmatisation [21]. We will argue that a COVID-19 label created a spoiled identity for patients in the community, as community members viewed COVID-19 patients as a potential source of disease and suffering in their communities.

## Materials and methods

### Study design

We conducted a qualitative study to explore the lived experiences of COVID-19 patients managed at home in Uganda.

### Study setting

The study was carried out among patients who reported to three hospitals that had been selected as treatment sites for COVID-19 patients in Uganda. One public hospital (Jinja Regional Referral Hospital) and two faith-based hospitals (Mengo Hospital and Nkozi Hospital). Mengo Hospital is classified as a Private Not for Profit (PNFP) hospital within the Ugandan healthcare system. It is one of the oldest hospitals in the country, having been established by missionaries in 1897 and is still a missionary hospital. Mengo has a bed capacity of over 300. The COVID-19 treatment unit (CTU) at Mengo Hospital is a 48-bed unit accredited by the Ministry of Health (MOH) to provide treatment for moderate and severe cases of COVID-19. The hospital started as a holding centre for COVID-19 patients in August 2020; however, active treatment of COVID-19 patients started in January 2021. The treatment of COVID-19 is at a fee.

Nkozi Hospital is also a missionary-founded PNFP hospital under the mandate of the Kampala Archdiocese. It operates for a period of 24 hours daily while offering curative, preventive, promotive and referral services both at the static and in outreaches. It has both inpatient and outpatient services. It has a 100-bed capacity. Treatment of COVID-19 is free of charge.

Jinja Regional Referral Hospital is located in Jinja City, approximately 84km East of the capital city, Kampala, Eastern Uganda. It has a bed capacity of 600 and serves as a referral hospital for ten districts. Treatment of COVID-19 is free of charge.

### Study population

COVID-19 patients managed at home from the three selected hospitals mentioned in the study setting were followed up using telephone interviews. We included patients who had tested positive for SARS-COV-2 but were not admitted to the hospitals but rather referred for home care. We excluded participants who were too sick to hold a telephone interview. Twenty participants were purposively selected to explore their experiences while being managed at home.

### Participant selection

Participants who had undergone home-based care were selected as follows: 7 participants from Jinja Regional Referral Hospital, 7 participants from Nkozi Hospital and 6 participants from Mengo Hospital. A list of all patients that had been registered at the COVID-19 treatment units in the three hospitals was obtained from the head of the unit. The unit head assisted the research assistant in identifying participants to contact for the study. The characteristics of these participants are presented in Table 1 below. Participants were recruited until the point of saturation when no new themes emerged. Participants were recruited until the point of saturation when no new themes emerged.

**Table 1:** Characteristics of patients that had undergone home-based care.

Characteristic	Frequency	Percentage
**Study centre**		
Mengo Hospital	6	30.0
Nkozi Hospital	7	35.0
Jinja Regional Referral Hospital	7	35.0
**Sex**		
Male	9	45.0
Female	11	55.0
**Age (years):** 34.5 (IQR = 16)		
**Religion**		
Christian	17	85.0
Muslim	3	15.0
**Marital status**		
Married	14	70.0
Single	6	30.0
**Education level**		
None	4	20.0
Primary	3	15.0
Secondary+	13	65.0
**Vaccination status**		
None	6	30.0
Only first dose	4	20.0
First and Second dose	10	50.0

### Data collection

During an initial in-depth interview, participants were informed of the purpose and procedure of this study, and after expressing willingness to participate, verbal consent was obtained, and an appropriate time scheduled for the interview was agreed upon. The interviews followed a semi-structured interview guide (included in S1 File) and were approximately 30 minutes long. Authors D.M and V.M, who are experienced in conducting qualitative interviews, and fluent in the local languages, conducted the interviews. Each interview was guided by a standardised procedure (introduction, interview, and end). First, participants were asked questions about their age, sex, marital status, educational level, religion, occupation and COVID-19 vaccination status. Participants were then invited freely to express their feelings and experiences. The order of the questions asked was flexible and adjusted according to the specific situation during the interview. With the permission of the patients, all the telephone-based interviews were audio recorded on the mobile phone and transcribed verbatim by the interviewer. The interviewer used a smartphone with the capability to record these interview sessions. The interview guides were used flexibly and modified according to the preliminary findings and as the need arose in the course of the study.

### Data analysis

The data were analysed using thematic content analysis. The first step was to read the transcripts repetitively to become immersed in the data, familiar with them and gain a full understanding of the interview data. The second step was to identify meaningful statements from phrases and sentences to generate the initial codes. Through this step, all actual data extracts were coded. Next, these meanings and initial codes were then extracted and placed into a table format to collate each code by analysing their relevance of them. Once the data had been sufficiently coded, the third step was to identify potential themes and sub-themes by combining all the relevant codes and data extracts into categories. The fourth step was to review and modify themes by comparing the external heterogeneity between themes and internal homogeneity within the themes, including the level of coded data extracts and the entire data as a whole. The final step was to prepare the report by selecting vivid examples (Table 2). The quote examples were reported in participants’ own words without any change to ensure precision. We used Nvivo 12.0.0 (QRS International, Cambridge, MA) to organise the analysis process.

**Table 2:** Examples of meaning units, codes, categories and themes from thematic content analysis of interviews about experiences during COVID-19 home-based care.

Meaning unit/quote	Code	Category	Theme
*I even felt like committing suicide because I had pain*, *{R16}*	Depression	Personal experiences as COVID-19 patients	Experiences of COVID-19 patients managed at home.
*My children and I have a very special attachment*, *and I felt sad that it was going to be broken soon by death*. *{R16}*	Fear		
*The thing that actually freaked me out most was I thought everyone was going to segregate me and that everyone would run away from me {R15}*			
*Automatically*, *they were in fear that I would infect other people at home or others if they visited {R5}*			
*My biggest fear was the disease becoming severe {R11}*			
*I thought that I would lose my job*. *I was going to be off work for two weeks {R14}*			
*I feared and thought she would miscarriage {R12}*			
*We would try everything as long as you’ve been advised or heard that others are using them*. *{R10)*	Desperation		
*I won’t lie to you; I made my will because I knew I was gone*. *{R16}*	Hopelessness		
*You can imagine the frustration*. *I have no child*, *no boyfriend no one living with me to comfort me*. *It was just me {R3}*	Loneliness		
*I would boil herbs*, *orange leaves*, *mango leaves and anything else I would imagine*, *like ginger and give them to steam*. *(R18)*	Complementary medicine		
*I think they should have prepared people before getting results*. *(R20)*	Counselling, home visits	Recommendations	
*Maybe the health workers should have visited to know how*			
*we are fairing*, *but they did not even call*. *(R18)*			
*I had this friend who would visit and drop something at the door and run away*, *then she would call or text to inform me {R3}*	Stigma	Experiences related to interaction with family/communities	

### Ethical considerations

Ethical approval to conduct the study was obtained from the Mengo Hospital Research and Ethics Committee, approval number MHREC-47/07-2021 and the Uganda National Council for Science and Technology (UNCST) with reference number HS2090ES. We also sought administrative clearance from the three hospitals that took part in the study. An initial in-depth interview was conducted where participants were informed of the purpose, procedure, and voluntary nature of the study. Verbal consent was obtained from all participants to participate in the research and for the interview to be audio recorded. We ensured confidentiality by removing identifiable information from the transcripts and using serial numbers instead of names, e.g. (Nkozi1, Nkozi2, Nkozi3). All recorded conversations were deleted from the smartphone after transferring them to a password-protected computer.

## Results

### Participant characteristics

A total of twenty participants were recruited; six were from Mengo hospital records, seven were from Nkozi hospital records, and seven were from Jinja regional referral hospital records. Participants’ ages ranged from 18 to 52 years. Overall, 60% of the participants were females, 85% were Christians, 70% were married, and 55% had attained a tertiary level of education (Table 1).

### Experiences of COVID-19 patients managed at home in Uganda

Our findings are organised into four main sections: personal experiences as COVID-19 patients, experiences related to interaction with family/communities, coping mechanisms, and recommendations. Table 2 summarises the process of coding, categorisation and generation of themes from patient quotes during the interviews.

#### Personal experiences as COVID-19 patients under home care/isolation

*"I wrote my will because I knew I was going to die"*: *Fear*, *Anxiety*, *and Hopelessness*. Participants reported feelings of fear. The fear was primarily a result of the false belief in their communities that all COVID-19 patients died as one participant told us: "*With all the stories that were going on about COVID-19*, *who wouldn’t fear*?*" (Nkozi 1*, *a 43-year-old*, *Male)*. Another Participant mentioned: "*I thought I was going to die*. *I knew that everyone that was testing positive for COVID-19 wasn’t surviving" (Nkozi 7*, *23-year-old*, *Female)*. Another participant mentioned: *I will not lie to you; I made my will because I knew I was gone*. *I even called my friend William and confessed that I was not surviving this because I was in too much pain*, *stressed and wasn’t getting any better (Mengo 2*, *32 years*, *Male)*. Since COVID-19 was of sudden onset, many of the participants were not prepared for this existential feeling and did not know how to cope with it. A 30-year-old female participant mentioned: "*I knew I was gone*, *I was trembling*, *I broke down*, *I was uncontrollably crying*. *At the time*, *once one tested for COVID-19*, *all that people would think about was death*. *The moment you tested positive for COVID-19*, *the next thing is death… Imagine dying with no child*. *It was so stressful*. *I would imagine people mourning*. *I had given up*, *especially when I had difficulty breathing" (Jinja 3*, *30 years*, *Female)*. Caregivers and relatives compounded the feeling of fear felt by the participants: "*My children almost fainted*, *they were uncontrollably crying*, *my mother was so scared because I am the breadwinner both at home and my family" (Jinja 2*, *48 years*, *Female)*. Furthermore, there was the fear of infecting loved ones: "*I thought they would infect the rest of the family members*. *That was scary but somehow*, *God protected us*.*"* In addition, participants feared the loss of jobs: *"I also thought that I would lose my job because I was going to be off work for two weeks (Nkozi 7*, *23 years*, *Female) "*. Participants also feared side effects associated with COVID-19, such as the loss of libido. The fear and anxiety resulted in a state of desperation, and many of the participants resorted to herbal medications: "*I would do anything I would hear of people testifying about or whatever I thought would help like I had heard that it doesn’t like heat so that is why I was steaming (Jinja 2*, *48 years*, *Female)*.*"*

"*I wanted to kill myself"*: *Loneliness*, *hopelessness and depression*. Participants reported feelings of loneliness, hopelessness and depression. The loneliness was primarily a result of the isolation measures imposed on those who had tested COVID-19 positive to prevent them from infecting others. One participant told us: "*Having been isolated in my house*, *sometimes I would want to interact with my children and husband*, *but there was no way" (Jinja 2*, *48 years*, *Female)*. Another told us: "*You can imagine the frustration; I have no child*, *no boyfriend*, *no one living with me to comfort me*, *it was just me" (Jinja 3*, *30 years*, *Female)*. Because of space limitations, many were confined in one place: *"I would also be bored and get tired of being in one place" (Nkozi 2*, *35 years*, *Male)*. There was also a feeling of hopelessness, depression and some even considered the option of suicide; one participant mentioned to us: *I think I had lost my senses because I was taking sick decisions*, *and I even felt like committing suicide because I had pain*, *was spending too much money and our government was getting money for its own things*, *yet we were paying so much in private hospitals that were helping (Mengo 2*, *32 years*, *Male)*. Another participant mentioned: *there is a way I used to feel that I just cannot explain*. *You hate yourself; you feel tired of everything around you and also have no appetite (Nkozi 1*, *43 years*, *Male)*. Some abandoned medication and therapies because of this feeling: *And then for me*, *after coming back from the hospital in Gayaza*, *I never steamed because I was tired of life*. *I was disgusted with life*, *and even when I would hear anyone laughing*, *I would feel bad*. Insensitive questioning from friends and family exacerbated the feelings: *people would question me in weird ways*, *and at a point*, *I would never feel like talking to anybody*. *I was disgusted with everyone*. *I just wanted to be alone because every time I moved out*, *people were acting horribly towards me (Jinja 3*, *30 years*, *Female)*. Furthermore, the feeling of loneliness and abandonment also made the depressive symptoms worse: *It was so challenging I felt like everyone had abandoned me*.

"*The flu wasn’t getting any better*. *It was tough"*: *Worsening symptoms of COVID-19*. Participants reported suffering from various symptoms such as cough, difficulty in breathing, loss of appetite, and weakness which often worsened during home care: *On the fourth day*, *I asked my husband*, *"How come you’re not getting any better*? *The cough is just increasing" I also told him that I was also feeling feverish*, *but I didn’t have a cough*. *He took it lightly (Mengo 3*, *23 years*, *Female)*.

Some participants reported that some symptoms are still present to this day: *my head pains terribly some evenings*, *that I develop a cold*. *I feel pain when I sit for long or stand*, *and I never had such issues before I was diagnosed with COVID-19 (Mengo 2*, *32 years*, *Male)*. Another participant mentioned: *I still feel pain in my chest*, *and I don’t know why or maybe the effects of the issue I developed with my lungs*. *I also sweat in my feet and hands*. *I never had that issue*, *but I am planning to go to the hospital to find out what could have caused it (Mengo 2*, *32 years*, *Male)*.

Many participants stated that the symptoms they experienced were the worst feelings they had ever had: *COVID-19 is a serious disease*. *I can’t even wish it on my worst enemy (Nkozi 5*, *23 years*, *Female)*.

*Use of complementary medicines*. Participants often complemented prescribed medicines with various herbs that were mostly inhaled during steaming: "*I would do anything I would hear of people testifying about or whatever I thought would help like I had heard that it doesn’t like heat*, *so that is why I was steaming (Jinja 2*, *48 years*, *Female)"*. Examples of substances used for steaming included benign and probably harmless substances such as lemon, ginger, and mango leaves but also possibly harmful herbs such as marijuana. These could sometimes help relieve some of the signs and symptoms of COVID-19. These substances were often supplied by caregivers, as an 18-year-old female told us: *"At a point*, *I would be breathing badly like I want to suffocate*, *and every time it happened*, *I would call mom*, *and she would bring me water with herbs for steaming then it would reduce*.*" (Mengo 1*, *18 years*, *Female)*. Some participants ascribed their improvement to complementary medicines: *"At first*, *I was taking only the prescribed treatment*, *but I wasn’t getting well*, *but when I started taking the warm water with ginger and lemon*, *I started feeling better even after steaming*. *I decided to take everything I was being advised to take" (Jinja 6*, *26 years*, *Female)*. Another participant told us: "*I was using juice made out of marijuana*, *lemon*, *ginger and other herbs I don’t know (Nkozi 6*, *38 years*, *Male)"*. One reason participants took the complementary medicine was because of anxiety and desperation: *I would do anything I would hear of people testifying about or whatever I thought would help like I had heard that it doesn’t like heat*, *so that is why I was steaming (Jinja 2*, *48 years*, *Female*).

Another reason that patients resorted to complementary medicine was that their symptoms were not abating with the conventional remedies.

"*How can I afford a balanced diet and yet I can’t afford the drugs*?*"*: *Economic burden associated with COVID-19 care*. Participants felt burdened by the cost of COVID-19 care. Many of the treatments prescribed were not provided by the government and patients bought them from private pharmacies. One participant noted that:

"*I sold my car because I was spending so much and I had no money to continue treatment yet; I also had to take care of my family bills*. *It was tough for me (Mengo 2*, *32 years*, *Male)*.*"* Some participants opted for home-based care due to the high cost of COVID-19 care in hospitals as one participant mentioned: "*I wouldn’t stay in hospital because there was no money*, *so I asked if I could isolate myself from the rest of the family members from home (Nkozi 2*, *35 years*, *Male)"*. The economic burden was compounded by the fact that many workers had lost their jobs: *And during that time*, *we were not working because*, *for me*, *I work in a training institute and at the time*, *schools were closed*, *and we didn’t have money yet were spending a lot of money*, *buying sanitisers*, *masks and treatment…*. *the health workers would tell us to eat well*, *but how can I afford a balanced diet and yet I can’t afford the drugs*? *(Jinja 2*, *48 years*, *Female)*. Some participants almost failed to foot their bills: *I was so scared*, *like you are when you don’t have money*. *Indeed*, *they asked me for the money that I didn’t have*. *I informed my sisters and brothers*, *and they contributed money*, *and I cleared the bill (Nkozi 5*, *23 years*, *Female)*.

The economic burden persisted even after the illness, as many were left in debt:

We got loans to cope with the financial status because little money was coming in, and up to now, we’re still affected because we have to pay the loans (Mengo 5, 40 years, Female)

#### Experiences related to interaction with family/communities

*"My friend would drop food at the door and run away"*: *Stigma associated with COVID-19*. COVID-19 patients experienced various forms of stigma. Participants were avoided majorly because of the fear of contracting the disease. One of the participants mentioned: "*When I reached home*, *people were running away from me*. *They feared me*. *They didn’t want to talk to me because they thought I was going to infect them with COVID-19" (Nkozi 7*, *23 years*, *Female)*. Participants also reported rejection from friends: "*Even my friends didn’t want to come near me*, *they would say*, *"You have COVID-19"*, *and they would only talk to me on phone*. *You would want to talk to people*, *but they wouldn’t accept because you have COVID-19" (Jinja 2*, *48 years*, *Female)*. In addition, participants also experienced stigma by people in the community: "*Meanwhile*, *it was worse with the community*. *Hoooh*! *In my area*, *when they heard that I had tested positive for COVID-19*, *they would advise other people not to come to my home*, *"don’t go to his home*, *they have COVID-19" if any dared to come*, *they wouldn’t want that person to associate with them because they would assume that I have infected the person and they would die*. *Sometimes I would move out of the house*, *and if someone passed by my home and I greeted them*, *they would quickly pass like they’ve not seen me*. *It was so bad*. *People would run away saying*, *"he has Covid*, *he has Covid"*. *I only decided to stay home*. *My wife would go buy things if we needed any*, *but if it was me*, *people wouldn’t touch my money*. *They would run saying that I would infect them*, *and they die*.*" (Nkozi 5*, *23 years*, *Male)*. An 18-year-old female participant also stated: *"We were going to the shop and someone shouted from our neighbourhood that*, *"abakovidi babo" meaning that we are the COVID-19 patients and everyone looked at us*.*" (Mengo 1*, *18 years*, *Female)*.

The stigma also had economic consequences: *I had two in-laws that lived with us at my home*. *They used to commute from my home to work in the market*. *They were chased from the market*, *and they were asked to provide negative results before they could go on with their work*. *These are women who couldn’t even afford the test (Nkozi 6*, *38 years*, *Male)*.

Participants also felt stigmatised while seeking care at the hospital. One participant stated that: "*Let me tell you something that got hair off my head*, *something that scared me most*. *When I had just entered the hospital*, *I coughed*. *You know it can be hard not to cough when you have cough*, *and some female health worker rudely sent me out*,*" Go*, *go out" (Mengo 3*, *23 years*, *Female)*. Another participant stated that: *"Doctors would come periodically and briefly then leave*. *You would need to talk to the doctor*, *but even the doctors were scared to get in contact with us" (Jinja 7*, *52 years*, *Male)*.

#### Coping mechanisms

Participants relied on specific behaviours and strategies that would make them feel more relaxed and cope with the disease.

*"I even had to give my life to Christ because it was too much"*: *Recourse to GOD*. Some participants tried to cope with the disease by praying, reading the bible, fasting and being thankful for God’s blessings. One participant said: "*I even had to give my life to Christ because it was too much*. *I used to pray; if possible*, *I would fast also*. *All I wanted was my family to be fine" (Mengo 3*, *23 years*, *Female)*. Another participant mentioned that: *"when we got COVID-19*, *I was only praying to God*. *I prayed for his protection because I thought we were going to die*. *When I spoke to God and read the bible*, *I would find comfort in God’s word*, *and I knew we would be healed" (Mengo 5*, *40 years*, *Female)*.

*Engaging in fun activities and entertainment*. To cope with the anxiety caused by COVID-19 and staying at home in isolation, participants engaged in fun and entertaining activities such as indoor exercises, jogging and walking around, dancing and listening to music, and communicating virtually through social media and weeding. These would help them reduce feelings of loneliness and anxiety. A participant told us:

*"I was doing some indoor exercises like skipping from my house*. *I would also dance and sometimes walk around" (Jinja 6*, *26 years*, *Female)*. Another participant told us:

"*I had a phone so I could play music and dance*, *mom never refused me to dance*, *so that kept me busy*. *I would play games on my phone*, *chatting with my friends" (Mengo 1*, *18 years*, *Female)*.

Another participant told us:

"*I was doing press-ups*, *jumping on a rope*, *I would also run very early in the morning before people would wake up to stress me*. *I would also feel better being on social media" (Nkozi 7*, *23 years*, *Female)*.

Economically, participants coped by taking loans, borrowing from family members and sometimes workmates: *we got loans to cope with the financial status because little money was coming in and up to now*, *we’re still affected because we have to pay the loans (Mengo 5*, *40 years*, *Female)*.

#### Participant’s recommendations to improve home care

Suggestions to improve COVID-19 care voiced by the participants included: counselling before and after testing, provision of free medical care, including at private facilities, and follow-up by health workers.

The need for counselling before and after testing for COVID-19 and during the treatment and recovery period was one of the strategies mentioned by participants:

"*They should have first prepared us before testing us or before giving us results*, *but they were just giving us results without any preparations*. *You see*, *before one tests for HIV/AIDS*, *they’re counselled*, *and they’re prepared for anything*. *It was a bit traumatising to be given the positive results before being prepared" (Jinja 6*, *26 years*, *Female)*.

Another participant noted that counselling should not only be done during the illness but even after recovery since many people remain psychologically scared: "*I think they should organise counselling sessions for people who had COVID-19 because people are traumatised" (Jinja 2*, *48 years*, *Female)*.

Another recommendation was the provision of COVID-19 care, services, treatment and drugs at no cost: *"I think drugs should have been provided free of charge because I can imagine how many people couldn’t afford the drugs" (Jinja 5*, *41 years*, *Male)*. Another participant mentioned: *"Most people died because they couldn’t afford the drugs*. *In case another pandemic comes*, *the government should prepare and give those affected free treatment to reduce on the high death rates" (Jinja 2*, *48 years*, *Female)*.

There was also a concern about the lack of adequate preparation for home-based care by the health services and the need to provide free logistical services such as free transportation back home for infected persons without private means: *"Maybe giving us treatment for free other than asking to buy and providing transport like an ambulance to the patients who test positive because we were mixing with other people in private means yet you have to go home*, *how did you expect us to travel*?*" (Jinja 1*, *42 years*, *Male)*.

Some participants suggested that follow-up visits by health workers would help improve COVID-19 management at home: "*Maybe they should have visited to know how we’re fairing*, *but they did not even call" (Mengo 4*, *41 years*, *Female)*. Another participant suggested that: *"Maybe the doctors should have checked on us because people didn’t like us in the village*. *Maybe the doctors would tell them that we’re now fine and we won’t infect them at all (laughs)" (Nkozi 5*, *23 years*, *Male)*.

## Discussion

Our study described the experiences of COVID-19 patients managed at home in Uganda. The findings are summarised into four content areas: personal experiences as COVID-19 patients, experiences related to interaction with family/communities, coping mechanisms, and recommendations.

### Experiences related to the individuals

COVID-19 patients managed at home experienced fear. Primarily, they feared death. This could be attributed to their perception that all COVID-19 patients died, the lack of an effective drug to treat COVID-19 at the time and the mass media reports of increasing COVID-19 deaths. In line with our findings, the fear and anxiety related to the ambiguity of the disease amongst COVID-19 patients were also reported in other studies [22–25]. Patients also experienced fear of infecting others, which could be attributed to the high contagion of COVID-19 and the scary disease management process when one contracted it. Caregivers of COVID-19 patients also compounded the fear. Caregivers feared their patients were going to die and their patients would either infect them or infect other family members. Our findings are consistent with the results of a study conducted among family caregivers of COVID-19 patients in Iran [26]. Participants also feared the side effects of COVID-19 disease, for example, loss of libido, and this could be attributed to the information given to them by other patients who suffered from these side effects or mass media misinformation.

Our study showed that patients managed at home experienced depression and loneliness as a result of the isolation measures imposed on COVID-19-positive patients to prevent infecting others. A study by Brooks suggests that isolation leads to negative emotional states such as irritability, insomnia, decreased concentration, anxiety and depression [27]. Depression among these patients could be attributed to the disease agony that they faced and the media reports of the increasing number of COVID-19 infections and deaths. A qualitative study by Zhong et al. revealed that excessive use of social media by COVID-19 patients was associated with severe symptoms of depression [28]. Our findings are consistent with qualitative studies conducted in Iran and China, where COVID-19 patients experienced fear, anxiety, hopelessness and depression [22, 23, 29]. Similarly, patients who suffered from other COVID-19-like diseases, such as Ebola, also reported unfavourable psychological experiences [30].

The participants reported a high economic burden associated with COVID-19 care. The high direct and indirect treatment costs were a challenge to most COVID-19 patients, and some opted to seek treatment from home to avoid these costs [31]. Participants had concerns regarding their jobs, some had lost their jobs, and others were worried about losing their jobs due to the uncertainty and the mounting economic challenges. A study by Khodabakhshi explored the economic concerns regarding COVID-19 and found that one of the major concerns was the subsequent financial consequences and problems [32]. In agreement with our results, occupational and financial concerns were reported in other studies conducted in Iran among COVID-19 patients [22, 23, 25].

### Experiences relating to their interaction with their families/communities

Patients mentioned social stigma multiple times even after full recovery, an experience that is more difficult than the physical pain of the disease. The social complications of stigma have a devastating effect on health; our results suggested that the stigma related to COVID-19 resulted in psychosocial and economic consequences. Other studies have also shown that stigma can affect COVID-19 patients’ lives socially, personally, and economically [22, 23, 25, 33].

The COVID-19 label on an individual can be seen as one that ’spoiled’ their identity and resulted in psychosocial and economic problems among COVID-19 patients. The feeling of fear, anxiety and depression could be explained by the theory of self or internal stigma. The internalisation of stigma triggered more social withdrawal as a strategy for patients to conceal their status and escape discrimination or other stigma enactments. Internal stigma could also have resulted from enacted stigma. Enacted stigma presented as interpersonal discrimination, labelling, gossiping about, and insulting of the patients. These experiences were prevalent in homes, hospitals and the community, and they caused anguish in the lives of participants. Similar incidents have been reported in prior studies on the experiences of COVID-19 patients [22, 23, 25].

### Coping mechanisms

Participants identified strategies to enable them to cope better with the disease. One of the strategies used by the participants to cope with the disease was spirituality. Participants were praying more, reading the bible, fasting and being thankful for God’s blessings. This could be attributed to the religious teachings to people in places of worship that by relying on God, they could overcome difficult situations. Studies have shown that during times of crisis and natural disasters, a closer relationship with God can facilitate withstanding the situation [34–36]. A study by Danhauer *et al*. found that spiritual-religious adaptation was one of the main ways used to cope with disease [37].

Participants maintained emotional and social support by communicating virtually using social media. Studies have shown that the use of social media can help improve the condition of COVID-19 patients through sharing information [38–40]. As social distancing is one of the standard operating procedures in the prevention of COVID-19 spread, people resorted to social media networks to compensate for the lost contact time with family and friends. They were able to remain in isolation and maintain their connection with the outside world.

Some COVID-19 patients were engaged in fun and entertaining activities. These activities included indoor exercises such as jogging, walking, dancing, and listening to music, and outdoor ones such as weeding. Doing fun and entertaining activities can be a tool against frustration and anxiety caused by COVID-19; it improves patients’ mental health and fastens the process of recovery and return to normal life. This makes the negative aspects of the disease less vital to them. In our study, another mechanism used by patients with COVID-19 to ease recovery was the use of complementary medicines. Similar findings were reported by a study conducted among COVID-19 patients in Iran [41].

### Recommendations

Our study showed that there is a need for counselling before and after testing for COVID-19 during the treatment and recovery period. COVID-19 patients experience negative emotions during the disease process and after recovery, thus the need for counselling. Our study revealed the need for the provision of COVID-19 care at a subsidised fee or at no cost. The high COVID-19 treatment costs were a big financial burden to the people living in a resource-limited country like Uganda.

### Strengths and limitations

This study focuses on a population that is often overlooked in hospital care, and this includes the outpatients or those patients who receive care while at home. The study was able to get a rich experience from the patient’s perspective. The major limitation was the study team was not able to physically interview participants. Telephone interviews may have limited participants’ expression as they sought to limit the length of the telephone call. At the time of the study, the ethics regulatory bodies discouraged physical meetings due to the risk of spreading or contracting COVID-19.

## Conclusion

COVID-19 home-based care in Uganda was associated with great fear, anxiety, loneliness, depression, economic loss and stigma from the community. Policymakers should consider various home-based follow-up strategies and strengthen counselling of COVID-19 patients at all stages of care.

## Supporting information

S1 Checklist COREQ (COnsolidated criteria for REporting Qualitative research) checklist. (PDF)

S1 File In-depth interview guide. (DOC)

## References

[1] Worldometer. COVID-19 Coronavirus Pandemic 2021 2023 [cited 2023 November 11]. Available from: https://www.worldometers.info/coronavirus/.

[2] Olum R, Bongomin F. Uganda’s first 100 COVID-19 cases: trends and lessons. Int J Infect Dis. 2020;96:517–8. doi: 10.1016/j.ijid.2020.05.073 32464272 PMC7247991

[3] Ministry of Health U. BASIC INFORMATION FOR COVID-19 PATIENTS UNDERGOING HOME BASED ISOLATION AND CARE 2020 [cited 2023 Jan 18]. Available from: https://www.unicef.org/uganda/reports/basic-information-covid-19-patients-undergoing-home-based-isolation-and-care.

[4] Migisha R, Kwesiga B, Mirembe BB, Amanya G, Kabwama SN, Kadobera D, et al. Early cases of SARS-CoV-2 infection in Uganda: epidemiology and lessons learned from risk-based testing approaches–March-April 2020. Globalization and Health. 2020;16(1):114. doi: 10.1186/s12992-020-00643-7 33239041 PMC7686950

[5] Bong CL, Brasher C, Chikumba E, McDougall R, Mellin-Olsen J, Enright A. The COVID-19 Pandemic: Effects on Low- and Middle-Income Countries. Anesthesia and analgesia. 2020;131(1):86–92. Epub 2020/04/04. doi: 10.1213/ANE.0000000000004846 ; PubMed Central PMCID: PMC7173081. 32243287 PMC7173081

[6] Fan G, Tu C, Zhou F, Liu Z, Wang Y, Song B, et al. Comparison of severity scores for COVID-19 patients with pneumonia: a retrospective study. European Respiratory Journal. 2020;56(3):2002113. doi: 10.1183/13993003.02113-2020 32675205 PMC7366179

[7] Li T, Hu Y, Xia L, Wen L, Ren W, Xia W, et al. Psychological experience of patients with confirmed COVID-19 at the initial stage of pandemic in Wuhan, China: a qualitative study. BMC Public Health. 2021;21(1):2257. doi: 10.1186/s12889-021-12277-4 34895189 PMC8665711

[8] Sahoo S, Mehra A, Suri V, Malhotra P, Yaddanapudi LN, Dutt Puri G, et al. Lived experiences of the corona survivors (patients admitted in COVID wards): A narrative real-life documented summaries of internalized guilt, shame, stigma, anger. Asian journal of psychiatry. 2020;53:102187. Epub 2020/06/09. doi: 10.1016/j.ajp.2020.102187 ; PubMed Central PMCID: PMC7261080. 32512532 PMC7261080

[9] Bologna L, Stamidis KV, Paige S, Solomon R, Bisrat F, Kisanga A, et al. Why communities should be the focus to reduce stigma attached to COVID-19. The American Journal of Tropical Medicine and Hygiene. 2021;104(1):39. doi: 10.4269/ajtmh.20-1329 33258438 PMC7790080

[10] Banerjee D. The COVID-19 outbreak: Crucial role the psychiatrists can play. Asian journal of psychiatry. 2020;50:102014. Epub 2020/04/03. doi: 10.1016/j.ajp.2020.102014 ; PubMed Central PMCID: PMC7270773. 32240958 PMC7270773

[11] World Health Organization. World Patient Safety Day 2023: Engaging Patients for Patient Safety: World Health Organization; 2023 [cited 2023 23rd May]. Available from: https://www.who.int/news-room/events/detail/2023/09/17/default-calendar/world-patient-safety-day-2023—engaging-patients-for-patient-safety.

[12] Richards T, Scowcroft H. Patient and public involvement in covid-19 policy making. BMJ (Clinical research ed). 2020;370:m2575. Epub 2020/07/03. doi: 10.1136/bmj.m2575 . 32611571

[13] Goffman E. 1963Stigma: Notes on the management of spoiled identity. New York: Simon & Shuster. 1963.

[14] Stigma Scambler G. and disease: changing paradigms. The Lancet. 1998;352(9133):1054–5. 10.1016/S0140-6736(98)08068-4 9759769

[15] Mahajan AP, Sayles JN, Patel VA, Remien RH, Sawires SR, Ortiz DJ, et al. Stigma in the HIV/AIDS epidemic: a review of the literature and recommendations for the way forward. AIDS (London, England). 2008;22 Suppl 2(Suppl 2):S67–79. Epub 2008/07/25. doi: 10.1097/01.aids.0000327438.13291.62 ; PubMed Central PMCID: PMC2835402. 18641472 PMC2835402

[16] Castro A, Farmer P. Understanding and addressing AIDS-related stigma: from anthropological theory to clinical practice in Haiti. American journal of public health. 2005;95(1):53–9. doi: 10.2105/AJPH.2003.028563 15623859 PMC1449851

[17] Kako PM, Dubrosky R. "You comfort yourself and believe in yourself": exploring lived experiences of stigma in HIV-positive Kenyan women. Issues in mental health nursing. 2013;34(3):150–7. Epub 2013/03/13. doi: 10.3109/01612840.2012.740765 . 23477435

[18] Kalichman SC. The harms of internalized AIDS stigma: a comment on Tsai et al. Annals of behavioral medicine: a publication of the Society of Behavioral Medicine. 2013;46(3):256–7. Epub 2013/07/12. doi: 10.1007/s12160-013-9529-z . 23843080

[19] Karamouzian M, Hategekimana C. Ebola treatment and prevention are not the only battles: understanding Ebola-related fear and stigma. International journal of health policy and management. 2015;4(1):55–6. Epub 2015/01/15. doi: 10.15171/ijhpm.2014.128 ; PubMed Central PMCID: PMC4289040. 25584356 PMC4289040

[20] Overholt L, Wohl DA, Fischer WA 2nd, Westreich D, Tozay S, Reeves E, et al. Stigma and Ebola survivorship in Liberia: Results from a longitudinal cohort study. PloS one. 2018;13(11):e0206595. Epub 2018/11/30. doi: 10.1371/journal.pone.0206595 ; PubMed Central PMCID: PMC6261413. 30485311 PMC6261413

[21] IFRC U. WHO. Social Stigma associated with COVID-19. A guide to preventing and addressing social stigma. Geneva: WHO Press; 2020.

[22] Alipour F, Arshi M, Ahmadi S, LeBeau R, Shaabani A, Ostadhashemi L. Psychosocial challenges and concerns of COVID-19: a qualitative study in Iran. Health. 2020:1363459320976752. doi: 10.1177/1363459320976752 33287564

[23] Jamili S, Ebrahimipour H, Adel A, Badiee Aval S, Hoseini SJ, Vejdani M, et al. Experience of patients hospitalized with COVID‐19: A qualitative study of a pandemic disease in Iran. Health Expectations. 2022;25(2):513–21. doi: 10.1111/hex.13280 34224643 PMC8444893

[24] Ahmadi S, Irandoost SF, Ahmadi A, Yoosefi Lebni J, Mohammadi Gharehghani MA, Baba Safari N. Explaining Experiences, Challenges and Adaptation Strategies in COVID-19 Patients: A Qualitative Study in Iran. Front Public Health. 2022;9:778026-. doi: 10.3389/fpubh.2021.778026 . 35186867 PMC8850373

[25] Zhang H, Xie F, Yang B, Zhao F, Wang C, Chen X. Psychological experience of COVID-19 patients: a systematic review and qualitative meta-synthesis. American Journal of Infection Control. 2022. 10.1016/j.ajic.2022.01.023 PMC8806710 35121042

[26] Nakhae M, Abolfazl Vagharseyyedin S, Mahmoudirad G, Shahdadi H. The experience of family caregivers in treatment of COVID-19 patients: a qualitative study. Neuropsychiatria i Neuropsychologia/Neuropsychiatry and Neuropsychology. 2021;16(3):131–7. doi: 10.5114/nan.2021.113313

[27] Brooks LM. The relation of spatial isolation to psychosis. The Journal of Abnormal and Social Psychology. 1933;27(4):375.

[28] Zhong B, Huang Y, Liu Q. Mental health toll from the coronavirus: Social media usage reveals Wuhan residents’ depression and secondary trauma in the COVID-19 outbreak. Computers in human behavior. 2021;114:106524. doi: 10.1016/j.chb.2020.106524 32836728 PMC7428783

[29] Wang C, Pan R, Wan X, Tan Y, Xu L, Ho CS, et al. Immediate psychological responses and associated factors during the initial stage of the 2019 coronavirus disease (COVID-19) epidemic among the general population in China. International journal of environmental research and public health. 2020;17(5):1729. doi: 10.3390/ijerph17051729 32155789 PMC7084952

[30] Chew QH, Wei KC, Vasoo S, Chua HC, Sim K. Narrative synthesis of psychological and coping responses towards emerging infectious disease outbreaks in the general population: practical considerations for the COVID-19 pandemic. Singapore medical journal. 2020;61(7):350. doi: 10.11622/smedj.2020046 32241071 PMC7926608

[31] Wasswa H. Covid-19: Uganda’s low inpatient numbers mask high community infection as desperate patients turn to herbs. BMJ (Clinical research ed). 2021;374:n1909. doi: 10.1136/bmj.n1909 34373252

[32] Khodabakhshi-koolaee A, Aghaei Malekabadi M. Motherhood and home quarantine: Exploring the experiences of iranian mothers in caring for their children during the COVID-19 outbreak. Journal of Client-Centered Nursing Care. 2020;6(2):87–96.

[33] Rahmatinejad P, Yazdi M, Khosravi Z, Shahisadrabadi F. Lived experience of patients with coronavirus (Covid-19): a phenomenological study. Journal of Research in Psychological Health. 2020;14(1):71–86.

[34] Glass K, Flory K, Hankin BL, Kloos B, Turecki G. Are coping strategies, social support, and hope associated with psychological distress among Hurricane Katrina survivors? Journal of Social and Clinical Psychology. 2009;28(6):779–95.

[35] Henderson TL, Roberto KA, Kamo Y. Older adults’ responses to Hurricane Katrina: Daily hassles and coping strategies. Journal of Applied Gerontology. 2010;29(1):48–69.

[36] Yoosefi Lebni J, Khorami F, Ebadi Fard Azar F, Khosravi B, Safari H, Ziapour A. Experiences of rural women with damages resulting from an earthquake in Iran: a qualitative study. BMC public health. 2020;20(1):1–13. 32375725 10.1186/s12889-020-08752-z PMC7201637

[37] Danhauer SC, Crawford SL, Farmer DF, Avis NE. A longitudinal investigation of coping strategies and quality of life among younger women with breast cancer. Journal of behavioral medicine. 2009;32(4):371–9. doi: 10.1007/s10865-009-9211-x 19308716

[38] Cuello-Garcia C, Pérez-Gaxiola G, van Amelsvoort L. Social media can have an impact on how we manage and investigate the COVID-19 pandemic. Journal of clinical epidemiology. 2020;127:198–201. doi: 10.1016/j.jclinepi.2020.06.028 32603686 PMC7320665

[39] Sharif N, Opu RR, Alzahrani KJ, Ahmed SN, Islam S, Mim SS, et al. The positive impact of social media on health behavior towards the COVID-19 pandemic in Bangladesh: A web-based cross-sectional study. Diabetes & Metabolic Syndrome: Clinical Research & Reviews. 2021;15(5):102206. doi: 10.1016/j.dsx.2021.102206 34298272

[40] Venegas-Vera AV, Colbert GB, Lerma EV. Positive and negative impact of social media in the COVID-19 era. Reviews in cardiovascular medicine. 2020;21(4). doi: 10.31083/j.rcm.2020.04.195 33388000

[41] Norouzadeh R, Abbasinia M, Tayebi Z, Sharifipour E, Koohpaei A, Aghaie B, et al. Experiences of Patients With COVID-19 Admitted to the Intensive Care Units: A Qualitative Study. Journal of Patient Experience. 2021;8:23743735211007359. doi: 10.1177/23743735211007359 34179418 PMC8205342

