# Supplementary material for: "I thought I was going to die": Experiences of COVID-19 patients managed at home in Uganda
Source: PLoS One. 2023 Dec 12;18(12):e0295113. doi: 10.1371/journal.pone.0295113 (PMC10715664; doi:10.1371/journal.pone.0295113)
Supplement: S1 File — (DOC) [file pone.0295113.s002.doc]

**In-depth Interview Guide:**

# Introduction and Oral consent

We are health workers at……………………………………..interested in exploring your experiences with COVID-19 and home care. We intend to to conduct a phone call that lasts approximately 40 minutes. We will not offer you any incentive or benefit for this call. The findings of this call will help to better the care of COVID-19 patients managed at home. All information obtained will be handled confidentially. Apart for a small possibility of breech of confidentiality, we don’t anticipate any other risk associated with this study. If you agree, we will record your phone call so that we go back to it to clarify some points. This recording will be kept confidentially. If you don’t agree we could simply have a telephone interview. There will be no consequences if you refuse to participate in this study any it will not affect any future care received. In fact, we will not write your name or personal identifiers. Kindly tell us if you agree to take part in this study?

# 1: Socio-demographic and interview information

| - 1. ID:   2. Interview date:   3. Interview start time:   4. Interview end time:   5. Place of residence:   6. Marital status:   7. Region:   8. Religion:   9. Education:   10. Main occupation: | - 1. Mother’s age:   2. Place of diagnosis of COVID-19:   3. Method of diagnosis:   4. Date of diagnosis:   5. Interviewer code:   6. Tape recording number: |
| --- | --- |

**2: Experiences with COVID-19**

2.1 Could you take us through your COVID-19 experiences?

Probe on feelings, fears, and thoughts

**3: Experiences with home care**

3.1 What went through your mind when you were told you were to be managed at home?

Probe: What were your biggest fears

3.2 What did your relatives think?

3.3 How did your neighbor’s react when they learnt you were being treated for COVID-19 at home?

3.4 What were you told to do at home?

3.5 What did you actually do?

3.6 What was the outcome of your disease?

3.7 What do you think the health workers could have done differently?

3.8 Any final thoughts?

Thank you so much.
